# Biochemical Identification of a Nuclear Coactivator Protein Required for AtrR-Dependent Gene Regulation in Aspergillus fumigatus

**DOI:** 10.1128/msphere.00476-22

**Published:** 2022-11-14

**Authors:** Sanjoy Paul, Shivani Ror, W. Hayes McDonald, W. Scott Moye-Rowley

**Affiliations:** a Department of Molecular Physiology and Biophysics, Carver College of Medicine, University of Iowa, Iowa City, Iowa, USA; b Proteomics Laboratory, Mass Spectrometry Research Center, Department of Biochemistry, Vanderbilt University Medical Center, Nashville, Tennessee, USA; University of Georgia

**Keywords:** ABC transporters, antifungal, *Aspergillus fumigatus*, azole, ergosterol, transcription factors

## Abstract

Azole drugs represent the primary means of treating infections associated with the filamentous fungal pathogen Aspergillus fumigatus. A central player in azole resistance is the Zn_2_Cys_6_ zinc cluster-containing transcription factor AtrR. This factor stimulates expression of both the *cyp51A* gene, which encodes the azole drug target enzyme, as well as an ATP-binding cassette transporter-encoding gene called *abcG1* (*cdr1B*). We used a fusion protein between AtrR and the tandem affinity purification (TAP) moiety to purify proteins that associated with AtrR from A. fumigatus. Protein fractions associated with AtrR-TAP were subjected to multidimensional protein identification technology mass spectrometry, and one of the proteins identified was encoded by the *AFUA_6g08010* gene. We have designated this protein NcaA (for nuclear coactivator of AtrR). Loss of *ncaA* caused a reduction in voriconazole resistance and drug-induced *abcG1* expression, although it did not impact induction of *cyp51A* transcription. We confirmed the association of AtrR and NcaA by coimmunoprecipitation from otherwise-wild-type cells. Expression of fusion proteins between AtrR and NcaA with green fluorescent protein allowed determination that these two proteins were localized in the A. fumigatus nucleus. Together, these data support the view that NcaA is required for nuclear gene transcription controlled by AtrR.

**IMPORTANCE** Aspergillus fumigatus is a major filamentous fungal pathogen in humans and is susceptible to the azole antifungal class of drugs. However, loss of azole susceptibility has been detected with increasing frequency in the clinic, and infections associated with these azole-resistant isolates have been linked to treatment failure and worse outcomes. Many of these azole-resistant strains contain mutant alleles of the *cyp51A* gene, which encodes the azole drug target. A transcription factor essential for *cyp51A* gene transcription has been identified and designated AtrR. AtrR is required for azole-inducible *cyp51A* transcription, but we know little of the regulation of this transcription factor. Using a biochemical approach, we identified a new protein called NcaA that is involved in regulation of AtrR at certain target gene promoters. Understanding the mechanisms controlling AtrR function is an important goal in preventing or reversing azole resistance in this pathogen.

## INTRODUCTION

Aspergillus fumigatus is the primary filamentous fungal pathogen of humans, and treatment of infections associated with this fungus is complicated due to problems with both diagnosis and limited antifungal therapies (discussed in references 1 and 2). A major complication arising with a troubling frequency is the appearance of azole-resistant A. fumigatus associated with aspergillosis (reviewed in reference 3). Infections caused by A. fumigatus strains that are azole resistant have a significantly increased rate of mortality (4), making the understanding of mechanisms underlying azole resistance a high priority.

The best-characterized mechanism of azole resistance in A. fumigatus involves linked changes in the gene producing the enzymatic target of azole drugs in this organism. This locus is called *cyp51A* and directs the production of the lanosterol α-14-demethylase enzyme, an essential step in the biosynthesis of ergosterol (5). Substitution mutations in the coding sequence of *cyp51A*, along with duplications in the promoter region of this gene, trigger a large decrease in azole susceptibility (6–8). These promoter duplications elicit increased levels of *cyp51A* gene transcription and are required for the relevant clinical phenotypes to be observed (9, 10). This critical link between transcription and drug resistance places a premium on analysis of the mechanisms underlying regulation of *cyp51A.*

Findings from several groups have implicated the A. fumigatus transcription factors SrbA and AtrR as essential contributors to expression of *cyp51A* (11–13). SrbA was discovered as a key regulator of gene expression in the ergosterol pathway based on its similarity to mammalian SREBP, which serves a similar function in control of cholesterol biosynthesis (14). AtrR was initially detected as a positive regulator of expression of ATP-binding cassette (ABC) transporter-encoding gene expression and was later shown to be important in transcription of both *cyp51A* as well as the ABC transporter-encoding locus *abcG1* (*cdr1B*/*abcC*) in A. fumigatus (12, 13).

While basic features of AtrR-responsive gene expression have been described, essentially nothing is known of how this factor is regulated, unlike the case for SrbA (15). To begin to investigate the modulators of AtrR transcription factor function, we prepared a functional fusion protein consisting of full-length AtrR fused at its C terminus to the tandem affinity purification (TAP) moiety. This AtrR-TAP fusion protein was expressed either from the native *atrR* promoter or from the strong *hspA* promoter. We prepared highly purified AtrR-TAP under gentle isolation conditions and analyzed the spectrum of copurifying proteins by using multidimensional protein identification technology (MudPIT) (16). Several proteins were identified that copurified with AtrR-TAP, and here we describe the characterization of a novel nuclear protein we have designated NcaA, for nuclear coregulation of AtrR.

## RESULTS

### Generation of the AtrR-TAP fusion strain.

**FIG 1:**
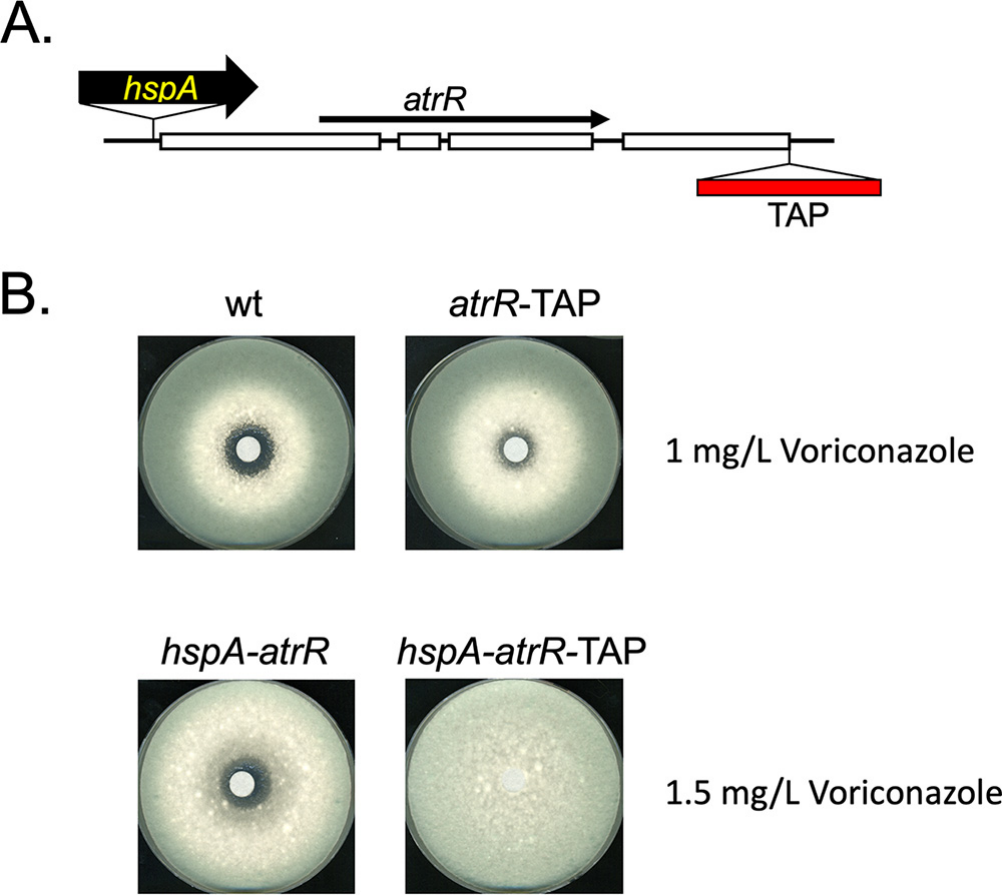
Characterization of *atrR*-TAP-expressing strains. (A) The alterations made to the *atrR* locus are diagrammed. The TAP tag cassette was inserted in place of the native *atrR* stop codon and expressed using either the native *atrR* promoter or with the A. fumigatus *hspA* promoter inserted immediately upstream of the native ATG codon. Exons and introns are indicated as open bars and solid lines, respectively. The transcriptional direction of *atrR* is indicated by the line. (B) Voriconazole susceptibility comparison of tagged and untagged *atrR* alleles. An equal number of spores of each indicated strain was plated on minimal medium and allowed to dry. A sterile filter disk containing the indicated dose of voriconazole was then placed in the center of the plate. Plates were incubated at 37°C and photographed.

To facilitate purification of AtrR from A. fumigatus cells, we constructed a fusion gene between *atrR* and a C-terminal tandem affinity purification (TAP) module that had been codon optimized for use in Aspergillus (17). We also fused this TAP moiety to a version of *atrR* in which the normal promoter had been replaced with the powerful *hspA* promoter (18). These constructs are diagrammed in Fig. 1A. We have previously found use of the *hspA* promoter to control *atrR* expression leads to overproduction of AtrR (12, 13). To test the function of these forms of *atrR*, we compared the voriconazole resistance phenotype of isogenic wild-type (wt) and *hspA-atrR* fusion genes with their TAP-tagged counterparts in a disk diffusion assay (Fig. 1B).

Introduction of the TAP tag module led to a decrease in voriconazole susceptibility in both the *atrR* and *hspA-atrR* formats. As we previously observed (12, 13) when a 3× hemagglutinin (HA) epitope tag was placed at the C terminus of AtrR, the TAP fusion proteins appeared to enhance the activity of the resulting AtrR fusion protein compared to the wild-type factor.

### Expression of TAP-tagged forms of AtrR.

Having confirmed that the introduction of the TAP moiety to the AtrR C terminus did not prevent function of the resulting factor, we assessed expression of these protein forms compared to that of the wild-type protein. Transformants expressing AtrR-TAP under the control of the wild-type *atrR* or *hspA* promoter were grown overnight, and whole-cell protein extracts were prepared. We analyzed isogenic versions of these strains lacking the TAP tag as controls. Equal amounts of extracts were analyzed by Western blotting using either anti-AtrR or anti-TAP antibodies.

**FIG 2:**
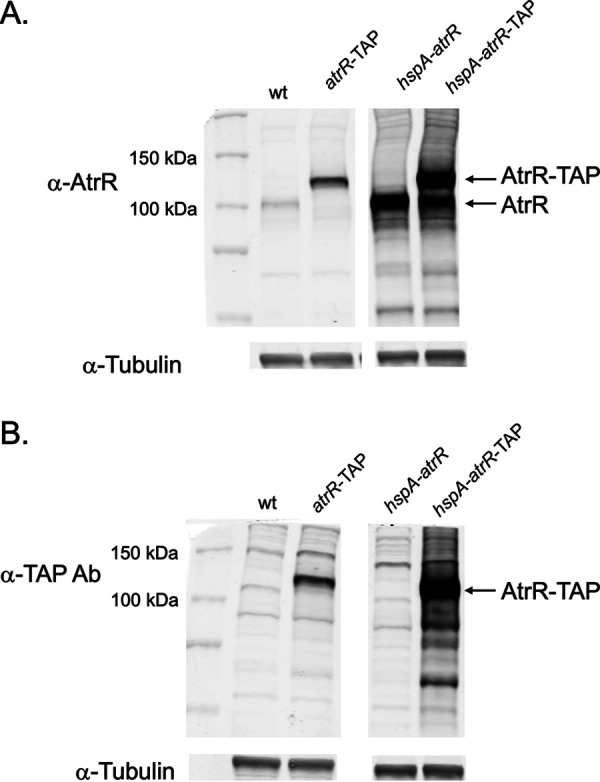
Western blot analysis of TAP-tagged *atrR* alleles. Whole-cell protein extracts were prepared from A. fumigatus strains containing the indicated forms of the *atrR* gene. Equal amounts of protein were resolved on SDS-PAGE, transferred to nitrocellulose filters, and probed with rabbit polyclonal antibodies directed against AtrR (α-AtrR) or the TAP moiety (α-TAP) or a mouse monoclonal antibody recognizing the tubulin protein (α-tubulin). (A) Results with anti-AtrR antiserum. Note the appearance of full-length AtrR in *hspA-atrR*-TAP strains. This is thought to be a result of proteolytic removal of the TAP tag. (B) Results with the anti-TAP antibody.

Expression of the AtrR-TAP fusion protein led to production of the expected higher-molecular-mass protein when driven by the wild-type *atrR* promoter, and the wild-type AtrR species was no longer visible (Fig. 2A). Based on the relative signals of AtrR and AtrR-TAP, the TAP fusion protein appeared to be expressed at a higher level, consistent with the decreased voriconazole susceptibility in this strain. Insertion of the *hspA* promoter upstream of either the wild-type *atrR* gene or the AtrR-TAP allele produced higher levels of each respective AtrR form. The presence of the *hspA-atrR*-TAP fusion produced both the higher-molecular-mass AtrR-TAP form as well as polypeptide that was close in size to that of the untagged AtrR. We suspect this may have resulted from proteolytic removal of the C-terminal TAP tag during the analysis.

These same protein extracts were probed with the anti-TAP antibody (Fig. 2B). A prominent polypeptide of 120 kDa was seen in both strains containing the *atrR*-TAP fusion gene, with the *hspA-atrR*-TAP strain producing higher levels of this protein. Some smaller polypeptides were also detected in this AtrR-TAP-overproducing strain, likely as a result of proteolysis. Only background signals were detected in the absence of the inserted TAP tag.

These data suggest that AtrR-TAP accumulates (at least under these conditions) as primarily a full-length protein and that the increased levels caused by TAP fusion might contribute to the increased level of voriconazole resistance seen in strains containing the AtrR-TAP fusion gene. To directly evaluate expression of AtrR target genes, we used Western blotting to examine levels of Cyp51A and AbcG1.

**FIG 3:**
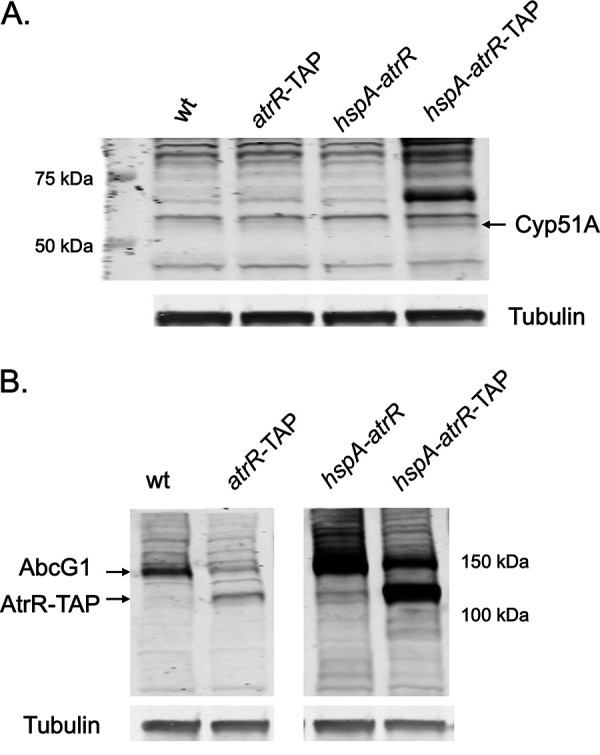
Function of TAP-tagged forms of AtrR. (A) Western blot analysis of Cyp51A expression in response to the presence of the indicated forms of the *atrR* gene. Whole-cell protein extracts were made from strains grown for 18 h. Equal aliquots of each extract were electrophoresed on SDS-PAGE and then analyzed by Western blotting using an anti-Cyp51A antibody (19). Location of the Cyp51A protein is indicated at the right. Tubulin was blotted to provide a loading control. (B) Western analysis of AbcG1 expression in strains with tagged or untagged forms of AtrR. The extracts described above were analyzed by Western blotting using a rabbit polyclonal antiserum directed against AbcG1 (32). Note that the ZZ domain present in the TAP tag was able to bind the Fc chain of immunoglobulins (20). This led to cross-reaction with the antibodies used to detect AbcG1.

We have previously described production of rabbit antibodies that can detect expression of the voriconazole target enzyme Cyp51A and the ABC transporter protein AbcG1 (19). Isogenic strains containing either *atrR* or *hspA-atrR* genes with or without a TAP fusion attached were grown overnight, and whole-cell protein extracts were prepared. These extracts were analyzed using either the anti-Cyp51A (Fig. 3A) or anti-AbcG1 (Fig. 3B) antibodies.

Expression of Cyp51A was only detected in the *hspA-atrR*-TAP strain. We had previously found that Cyp51A was undetectable using this Western blot assay in wild-type cells but could be induced by voriconazole induction or hyperactive promoter variants, such as TR34 or TR46 (8, 9). Here we were able to detect Cyp51A expression in the absence of any drug challenge in the presence of the *hspA-atrR*-TAP allele. This high basal level of Cyp51A in this strain may explain its elevated voriconazole resistance (Fig. 1B).

Western blotting for AbcG1 expression in these same backgrounds produced a distinctly different result. Expression of AbcG1 was higher in the presence of the untagged alleles of *atrR* compared to that of TAP-tagged versions (Fig. 3B). The presence of the *hspA* promoter led to increased AbcG1 expression compared to the same *atrR* protein form driven by the wild-type *atrR* promoter. Note the presence of cross-reaction of the TAP fusion proteins with the rabbit primary antibody, a well-known complication of this epitope tag (20).

The differential response of *abcG1* and *cyp51A* expression to these different forms of AtrR suggests a promoter-specific effect of this transcriptional regulator. Further studies, described below, support this suggestion.

### Purification of AtrR-TAP.

Having established that the AtrR-TAP fusion protein was able to function *in vivo* (albeit with some differences from the wild-type factor), we prepared native extracts and used standard TAP chromatographic approaches to purify this protein along with copurifying polypeptides. We employed multidimensional protein identification technology (MudPIT) to detect these copurifying proteins and found many different candidates (16). Here, we will focus on a single protein that copurified with AtrR-TAP and was 2 times more abundant in AtrR-TAP fractions from the *hspA*-driven fusion gene than when produced from the wild-type *atrR* promoter. This protein was designated NcaA, for nuclear coactivator of AtrR. NcaA is encoded by the gene *AFUA_6g08010*. While the predicted polypeptide produced by *ncaA* represented a previously undescribed protein, even though it is present in most fungal species, we provide evidence supporting the NcaA designation below.

### NcaA interacts with AtrR *in vivo*.

**FIG 4:**
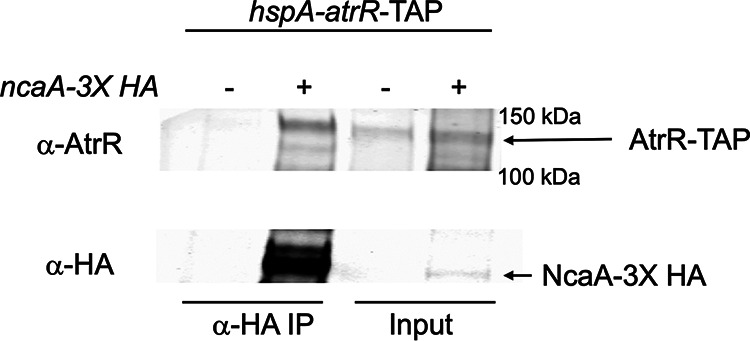
Association of NcaA and AtrR *in vivo*. A strain expressing AtrR-TAP and either containing (+) or lacking (-) an *ncaA*-3X HA fusion gene was grown for 18 h in Sabouraud dextrose medium. Whole cell protein extracts were prepared under native lysis conditions and used for immunoprecipitation with an anti-HA antibody. Samples of the native total lysate were retained to confirm the presence of each protein (Input). HA-immunoprecipitates were recovered and run in parallel followed by Western blotting with either anti-AtrR or anti-HA antibodies. The location of each protein is indicated at the right hand side.

To further support our identification of NcaA as an interactor with AtrR, we carried out a coimmunoprecipitation analysis using epitope-tagged forms of these two proteins. The *hspA-atrR*-TAP-containing strain was used to allow facile identification of AtrR, and a 3×HA-tagged form of *ncaA* was introduced into this strain. Isogenic *hspA-atrR*-TAP strains either containing or lacking the *ncaA*-3×HA allele were grown overnight, native protein extracts were prepared, and the NcaA-3×HA protein was recovered by immunoprecipitation with anti-mouse HA antibody. These anti-HA immunoprecipitates were electrophoresed on SDS-PAGE and then analyzed by Western blotting using either anti-AtrR or anti-HA antibodies (Fig. 4).

Only when both the NcaA-3×HA-tagged allele and the AtrR-TAP fusion were present was coimmunoprecipitation seen. Expression of only the AtrR-TAP fusion protein did not show any evidence for nonspecific recovery of this factor by the anti-HA antibody. These data support the view that AtrR and NcaA associate *in vivo*.

### NcaA is required for normal voriconazole resistance.

**FIG 5:**
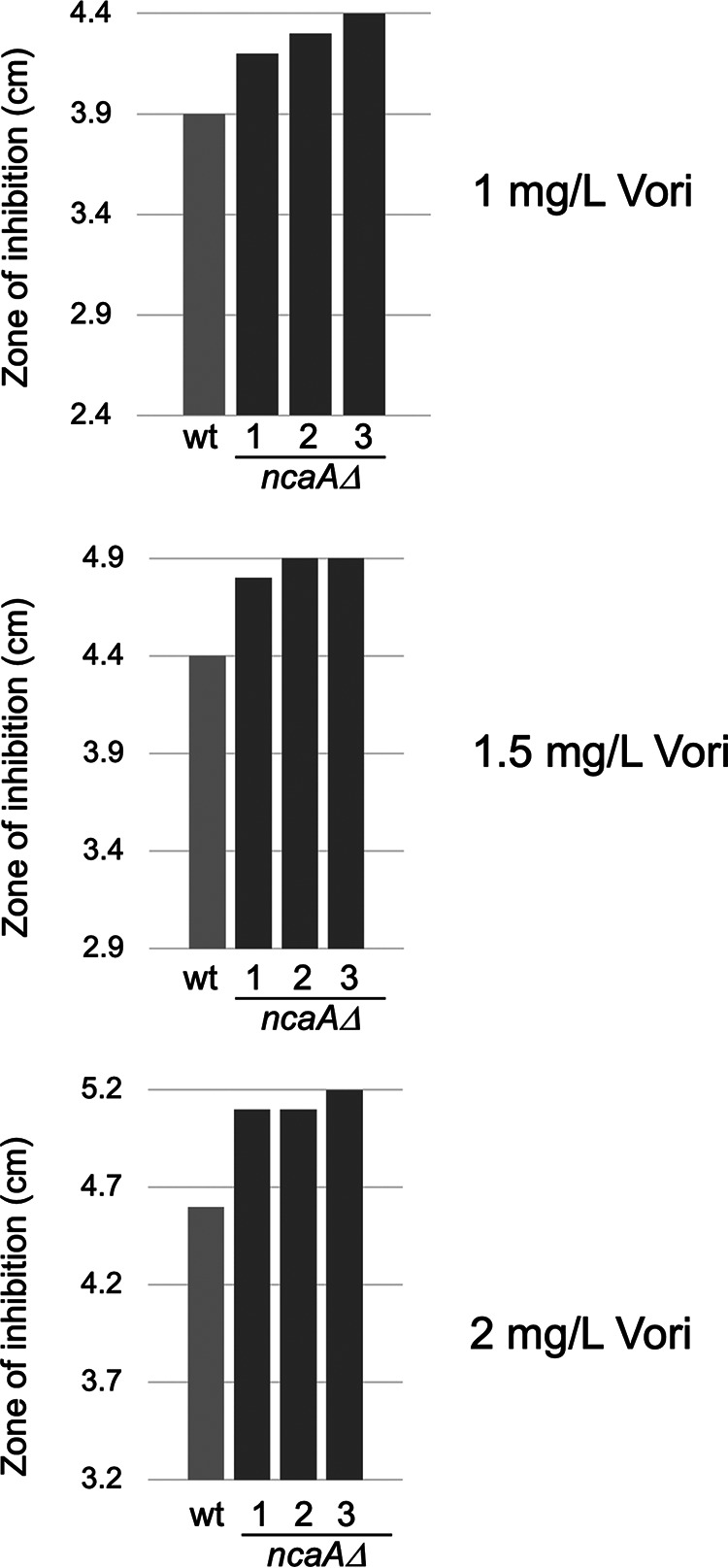
Loss of *ncaA* increased voriconazole susceptibility. Wild-type AfS35 cells and isogenic *ncaA*Δ mutants were grown and analyzed for voriconazole susceptibility using a disk-diffusion assay. Different doses of voriconazole were applied to each disk, and the plates were incubated at 37°C. The distance from the edge of the disk to the beginning of growth (zone of inhibition) was measured for 3 independent isolates of the *ncaA*Δ strain.

We generated a strain lacking the *ncaA* coding sequence using a CRISPR-based gene deletion strategy (21). Spores were produced from isogenic wild-type and *ncaAΔ* strains and plated on minimal medium. A filter disk containing different concentrations of voriconazole was placed in the center of these spores, and the resulting plate was incubated to allow growth of the cells. The distance from the disk at which growth stopped (zone of inhibition) was measured. These experiments were performed on three independent isolates of *ncaAΔ* (Fig. 5).

Loss of *ncaA* caused a modest but highly reproducible increase in voriconazole susceptibility. These data are consistent with NcaA playing a positive role in conferring voriconazole tolerance.

### NcaA is required for azole-induced expression of an AtrR target gene.

**FIG 6:**
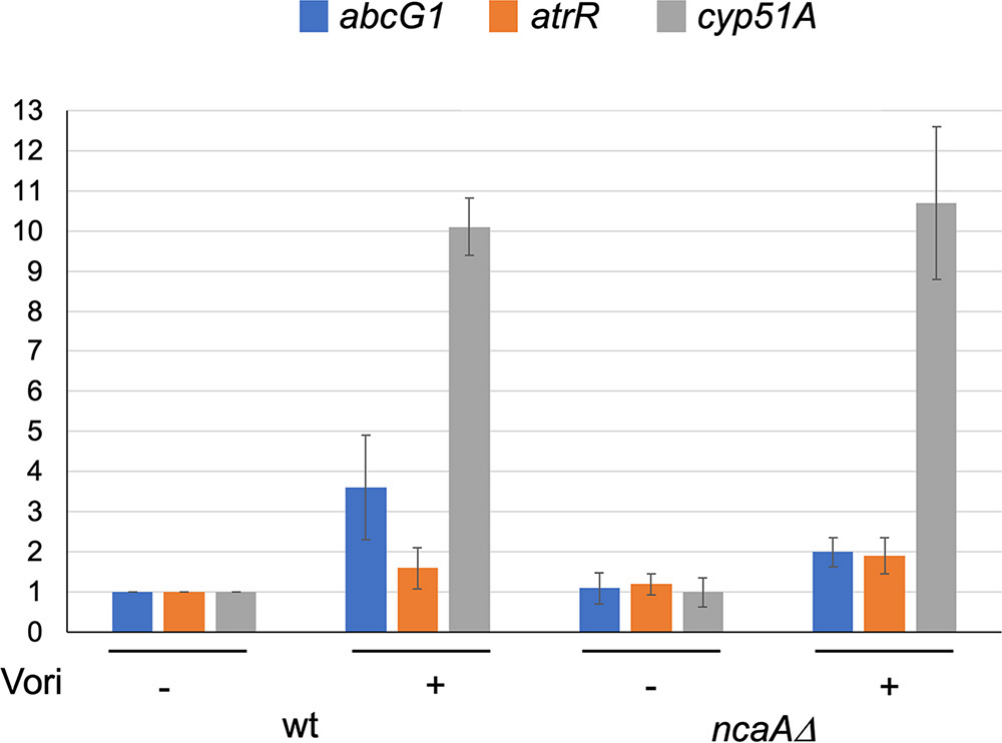
Absence of *ncaA* prevents normal voriconazole induction of *abcG1* expression. Isogenic wild-type and *ncaA*Δ strains were grown in the presence (+) or absence (−) of voriconazole. RNA was prepared from each culture, and steady-state mRNA levels of the indicated genes were measured using qRT-PCR.

To probe the requirement for NcaA in AtrR-dependent gene regulation, isogenic wild-type and *ncaAΔ* strains were grown overnight in the presence or absence of sublethal doses of voriconazole. These cultures were harvested, and total RNA was prepared and analyzed by quantitative reverse transcription followed by PCR analysis of three different mRNA species corresponding to known AtrR target genes. We used the *abcG1*, *cyp51A*, and *atrR* genes as representative AtrR-controlled genes (Fig. 6).

Transcription of *abcG1* was induced ~3.5-fold in the presence of voriconazole in wild-type cells, but this was reduced to ~2-fold in *ncaAΔ* strains. Loss of *ncaA* failed to impact expression of either *cyp51A* or *atrR*. This modest reduction in *abcG1* induction in the presence of voriconazole was consistent with the level of increased susceptibility seen earlier (Fig. 5).

### NcaA is localized to the nucleus.

**FIG 7:**
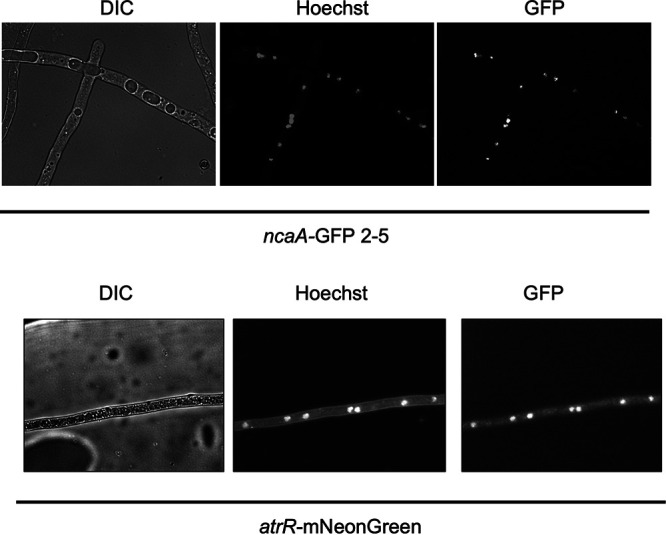
NcaA and AtrR are both localized to the fungal nucleus. A. fumigatus strains expressing either an *ncaA*-GFP fusion protein (top) or an *atrR*-mNeonGreen fluorescent protein (bottom) were grown overnight and then analyzed by light microscopy. Cells were visualized by Nomarski optics (differential interference contrast [DIC]), nuclei detected by staining with Hoechst dye. Nuclei and GFP were visualized with appropriate filters.

Based on its copurification with AtrR, we suspected that NcaA would be localized to the nucleus. We believed AtrR would be a nuclear factor, based on its clear role as a regulator of gene expression and ability to detect AtrR bound to its DNA target sites. To test these predictions, we constructed C-terminal fusion genes between *ncaA* and green fluorescent protein (GFP) as well as *atrR* with A. fumigatus codon-optimized mNeonGreen (mNG). Strains containing either the *ncaA*-GFP or *atrR*-mNG fusion genes were grown overnight and then visualized by microscopy (Fig. 7).

The NcaA-GFP fusion protein was found to be localized to the nucleus in A. fumigatus hyphae. Similarly, AtrR-mNG was also found in the nucleus. We confirmed the identity of the nuclear compartment by staining nuclear DNA with Hoechst dye. NcaA association with AtrR is likely to involve their association within this organelle.

## DISCUSSION

AtrR is a major determinant of azole resistance in A. fumigatus, but little is known of how this factor is regulated (12, 13). To identify proteins that may act to modulate AtrR function, we prepared and purified a TAP-tagged version of this transcription factor. We were able to detect a number of different proteins that copurified with AtrR-TAP; we have focused here on a previously uncharacterized protein we designated NcaA. NcaA is a novel protein with no obvious conserved structural domains. Analysis of the sequence of NcaA predicted a central region with coiled-coil domains (unpublished data) flanked by more disordered and flexible regions. Most Aspergillus species contain an ortholog of NcaA, suggesting that the function of this protein must be conserved across these related organisms.

Our data support a role for NcaA in transcriptional activation based on two different assays. First, loss of NcaA produced an increase in voriconazole susceptibility across a range of drug concentrations (Fig. 4). Second, a gene-specific defect in drug induction was seen for the AtrR target gene *abcG1*. Two other AtrR target genes were unaffected by loss of NcaA. The observation that drug induction of both *atrR* itself and the azole drug target-encoding gene *cyp51A* was unaffected is a likely a central factor in determining the resulting voriconazole susceptibility of the *ncaAΔ* strain. Our previous analyses of both the *abcG1* and *cyp51A* promoters may help explain the differential effects of the *ncaAΔ* allele on these two genes. Transcription of *abcG1* is highly dependent on AtrR activity, while expression of *cyp51A* involves both AtrR and also the key sterol regulatory transcription factor SrbA (10, 11, 14, 22, 23). NcaA may not be involved in AtrR-dependent activation at the *cyp51A* promoter, or the partially compromised phenotype triggered by the *ncaAΔ* allele may be suppressed by the presence of normal SrbA.

This gene-specific activation by AtrR was also seen in the characterization of the *atrR*-TAP allele. Cyp51A could be detected by Western blotting only in cells containing the *hspA-atrR*-TAP allele, not in cells expressing *atrR*-TAP from the native *atrR* promoter or *hspA-atrR*-containing strains. In contrast to the response of the *cyp51A* promoter, expression of the *atrR*-TAP fusion protein was generally less effective at driving transcription of *abcG1*. These data argue that while the presence of the TAP tag at the C terminus of AtrR prevents normal gene activation at the *abcG1* promoter, this same recombinant protein appears to be a more effective activator of *cyp51A*. We have previously documented that a 3×HA tag at the C terminus of AtrR behaves as a hypermorphic (activated) allele of *atrR* (13). Interestingly, the AtrR-3×HA construct seemed to be a better inducer of *abcG1* expression than *cyp51A.* The variable effects of these different AtrR fusion proteins suggest that the contacts made by this factor at different promoters are unique. Promoter-specific effects for transactivators have been documented before (24–27).

The nonidentical responses of AtrR target promoters to different AtrR fusion proteins illustrate the complexity of transcriptional activation by this factor. This is also likely to contribute to the phenotype caused by loss of NcaA. Purification of AtrR-TAP yields a population of complexes that represent an average of proteins associated with AtrR. Promoter-specific complexes may have formed that were recovered together during our purification. Further analyses are required to determine how the various factors that associate with AtrR contribute to the function of this protein and if these contribute equally at the various target promoters responsive to AtrR.

## MATERIALS AND METHODS

### A. fumigatus strains, growth conditions, and transformation.

The list of strains that were used in this study are listed in Table 1. A. fumigatus strains were routinely grown at 37°C in rich medium (Sabouraud dextrose; 0.5% tryptone, 0.5% peptone, 2% dextrose [pH 5.6 ± 0.2]). Selection of transformants was made in minimal medium (MM; 1% glucose, nitrate salts, trace elements, 2% agar [pH 6.5]; trace elements, vitamins, and nitrate salts were as described in the appendix of reference 28), supplemented with 1M sorbitol and either 20 mg/liter phleomycin (after adjusting the pH to 7) or 150 mg/liter Hygromycin Gold (both from InvivoGen). For solid medium, 1.5% agar was added. Doxycycline (Dox-off) promoter shutoff experiments were performed by adding 25 mg/liter doxycycline (BD Biosciences).

**TABLE 1:** A. fumigatus strains used in this study

Strain	Parent	Genotype	Source or reference
AfS35	D141	*akuAΔ::loxP*	FGSC
SPF148	AfS35	*atrR*-TAP::*hph*	This study
SPF108	AfS35	*ptrA-hspA-atrR*	13
SPF151	SPF108	*ptrA-hspA-atrR*-TAP::*hph*	This study
SPF151A	SPF108	*hspA-atrR*-TAP::*hph*	This study
SPF176	AfS35	*ncaAΔ*::*hph*	This study
SPF180	SPF151A	*ncaA*-3X HA::*ptrA*	This study
SRF-47	AfS35	*ncaA*-GFP2-5::*ble*	This study
SRF-50	AfS35	*atrR*-mNG::*ble*	This study

Generation of the *atrR*-TAP-tagged strain was done as follows. Plasmid pSP110 was constructed using Gibson assembly of 4 PCR fragments in a pUC19 vector: 1.2 kb corresponding to the 3′ end of the *atrR* gene, the G5 linker-TAP tag from plasmid pME4543 (from Bastian Joehnk and Gerhard Braus [see reference 18 for a description of the codon-optimized TAP cassette]), the transcription terminator-*hph* cassette from pSP98 (12), and a 1.2-kb region downstream of the *atrR* gene. To ensure accurate construction, pSP110 was sequenced to verify the integrity of the 3′ region of the *atrR* gene-G5 linker-TAP fusion present in the plasmid. This plasmid was cut with KpnI and HindIII restriction enzymes and transformed into either AfS35 or the SPF108 strain to generate *atrR*-TAP and *hspA-atrR*-TAP strains, respectively. Targeted integration of these strains was verified by PCR diagnosis of novel junctions formed downstream of the TAP tag, as well as by Western blotting of the strains with both AtrR and TAP antibodies. Transformation and generation of *ncaA*Δ mutants were performed using *in vitro*-assembled cas9-guide RNA ribonucleoproteins coupled with 50-bp microhomology repair templates (21). For deletion of *ncaA*, CRISPR RNAs (5′-CAGCTGTGACGCACAAGCGCGGG, corresponding to 5′ end of the gene and 5′-TACGCCCCAGAGCTAGGCGGTGG, corresponding to 3′ end of the gene) were used to replace *ncaA* with the hygromycin resistance marker cassette amplified from the plasmid pSP62 (13); we used ultramer-grade oligonucleotides from IDT (primer pairs ncaA-MH-Hph-F, 5′-TGCAATCTCAGGCCCCACTCTTCATCGTTCCAGCTGTGACGCACAAGCGCcctctaaacaagtgtacctgtg, and ncaA-MH-Hph-R, 5′-CGGCGGCTATCCGGTTTGGTAAGATCAAACTACGCCCCAGAGCTAGGCGGgatagctctgtacagtgaccg) harboring 50 bp of homology to the *ncaA* gene. In all primer pairs used for integration, targeted A. fumigatus DNA is in upper case, while PCR amplification primers are in lower case. The *ncaA*-GA5 linker-3×HA-tagged strain was generated using CRISPR RNA (5′-TACGCCCCAGAGCTAGGCGGTGG corresponding to 3′ end of the gene) to C-terminally tag *ncaA* with the 3×HA-pyrithiamine resistance marker cassette amplified from the plasmid pSP102, by using ultramer-grade oligonucleotides from IDT (primer pairs ncaA-MH-ptrA-F, 5′-GCCACCAGTCCCTTCCGGGTTCAAACTTGGCTAGTGCGTCTGGACCACCGCCTAGCTCTGGGGCGggtgctggtgccggt, and ncaA-MH-ptrA-R, 5′-TATGTAGGCTGATGGCGGCGGCTATCCGGTTTGGTAAGATCAAACTACGCatctgacagacgggcaattg) harboring 50 bp of homology to insert the GA5 linker-3×HA in place of the *ncaA* gene stop codon. The *ncaA-*GFP2-5 locus was constructed by using the pSR35 vector, which contains a codon-optimized GFP2-5 gene (29) for expression in A. fumigatus. The PCR amplicon was obtained with oligos ncaA-GFP2-5 MH F (CAGTCCCTTCCGGGTTCAAACTTGGCTAGTGCGTCTGGACCACCGCCTAGCTCTGGGGCGGGAGCCGGTGCCATGCC) and ncaA-GFP2-5 MH R (TCCTGAGGCCTACATATGTAGGCTGATGGCGGCGGCTATCCGGTCAGTCCTGCTCCTCGGC), which contained homologous regions before and after the stop codon of *ncaA*, the GFP2-5 tag, and phleomycin resistance gene. The AtrR protein was C-terminally tagged with A. fumigatus-optimized fluorescent protein mNeonGreen by using vector pSR25 (synthesized by GenScript, Piscataway, NJ). Primer pairs AtrR-CoNG MH F (CCCGGTCTTCGACACCAATGGTCCACCCCACGGTGGATTGGCTGGTGCCGGTGCTGGT) and AtrR-CoNG MH R (GCCCAAATAAGCCTCCCACGCTGGTGTCCGATTCGTTATTTCAGTCCTGCTCCTCGC) were utilized for amplification of a PCR product corresponding to a homologous region across the *atrR* stop codon, along with the mNeonGreen tag and phleomycin resistance marker gene. All the above C-terminally tagged strains were generated in the AfS35 background and genotypically confirmed by diagnostic PCR of the novel upstream and downstream junctions formed upon targeted integration. At least 3 independently targeted transformants were phenotyped for all *ncaA* mutants, of which a representative strain is depicted in the data presented. The list of strains used in this study is given in Table 1.

### TAP tagging.

AtrR was purified from *atrR*-TAP and *hspA-atrR*-TAP strains based on a protocol described preiously (30). Approximately 10^6^ spores of the TAP-tagged strains were inoculated in petri dishes containing 20 mL of Sabouraud dextrose broth at 37°C for 24 h. Mycelia that formed as a biofilm on the top were collected (~5 g) and ground into fine powder in liquid nitrogen using a mortar and pestle. The ground mycelium was resuspended in 10 mL B250 buffer (250 mM NaCl, 100 mM Tris-HCl [pH 7.5], 0.1% NP-40, 10% glycerol, 1 mM EDTA, 1 mM dithiothreitol [DTT], 1 mM phenylmethylsulfonyl fluoride [PMSF], as well as 200 mL of protease inhibitor cocktail for use with fungal and yeast extracts [Sigma catalog number P8215]) in ice-cold SS34 tubes and incubated for 10 min at 4°C, with intermittent vortexing of 30 s at setting 6 (Vortex Genie 2, Scientific Industries) every 2 min. The tubes containing resuspended mycelia were centrifuged at 25,000 × *g* for 30 min at 4°C. The supernatant was transferred to ice-cold 15-mL tubes containing 1 mL of IgG-Sepharose 6 Fast Flow (GE Healthcare) and incubated on a rotating platform for 3 h at 4°C. The crude extract–IgG-Sepharose suspension was poured into chromatography columns, and the extract was allowed to flow through with gravity. The IgG-Sepharose was then washed twice with 10 mL W250 buffer (250 mM NaCl, 40 mM Tris-HCl [pH 7.5], 0.1% NP-40, 1 mM DTT, 1 mM PMSF, as well as 100 μL of protease inhibitor cocktail [Sigma]), once with 10 mL W150 buffer (150 mM NaCl, 40 mM Tris-HCl [pH 7.5], 0.1% NP-40, 1 mM DTT), and finally once with tobacco etch virus (TEV) cleavage buffer (TCB; W150 buffer plus 0.5 mM EDTA). After the TCB wash, the chromatography column was resuspended with 1 mL TCB as well as 20 μL (200 U) of TEV protease enzyme (Genscript) on a rotator, and the columns were incubated at 4°C for 16 h. The TEV protease-treated suspension was then transferred into the new columns containing 500 μL Calmodulin-Sepharose 4B (GE Healthcare) and 6 mL of CBB buffer (150 mM NaCl, 40 mM Tris-HCl [pH 8.0],1 mM MgOAc, 2 mM CaCl_2_, 100 mM imidazole, 10 mM β-mercaptoethanol) and incubated on a rotating platform at 4°C for 1 h. At the end of incubation, the CBB was allowed to flow through. The column was then washed three times with 1 mL CBB (containing 0.02% NP-40). The proteins were extracted from the Calmodulin column by adding 1 mL EB buffer (W150 plus 20mM EGTA, 1 mM MgOAc, 0.02% NP-40, 10 mM β-mercaptoethanol) into the columns and incubating for 5 min at room temperature. The elution step was repeated again with 1 mL EB. The 2 mL of eluate was split into two 750-mL aliquots (for mass spectrometry analysis) and one 500-mL aliquot (for Coomassie and silver staining), precipitated in 25% trichloroacetic acid, and finally the pellet was washed with 1 mL cold acetone. The pellet was then stored at −70° C until use. MudPIT was performed, and the data were analyzed as previously described (31).

### Immunoprecipitation and Western blotting.

Approximately 10^6^ spores of the TAP-tagged strains were inoculated in petri dishes containing 20 mL of Sabouraud dextrose broth at 37°C for 24 h. Mycelium that formed as a biofilm on the top was collected (~500 mg) and was ground into a fine powder in liquid nitrogen using a mortar and pestle. The ground mycelium was resuspended in 1.5 mL B250 buffer in ice-cold 15-mL tubes and incubated for 10 min at 4°C, with intermittent vortexing of 30 s at setting 6 (Vortex Genie 2, Scientific Industries) every 2 min. The tubes containing resuspended mycelia were centrifuged at 5,000 × *g* for 10 min at 4°C. A 50-μL aliquot of the supernatant was kept aside as input control, while 750 μL was used for immunoprecipitation with HA monoclonal antibody 2-2.2.14 (Invitrogen) at 1:100 dilution; the mixture was incubated on a rotating platform for 16 h at 4°C. The cell lysate-antibody mixture was then added to 50 μL of protein G Dynabeads (Invitrogen) for 6 h on a rotator at 4°C. The cell lysate-antibody mixtures were washed with twice with W250 buffer and once with W150 buffer. The protein was eluted in 50 μL of 2× Laemmli sample buffer (Bio-Rad) after heating the Dynabeads at 95°C for 10 min. The input sample was also resuspended in 50 μL of 2× Laemmli sample buffer and incubated at 95°C for 10 min. A 20-μL aliquot of the input and immunoprecipitated sample was used for Western blotting, which was performed as described elsewhere (19). The AtrR polyclonal antibody used here has been described previously (13); it was used at a 1:500 dilution, while the TAP antibody (Genscript) and the HA monoclonal antibody 2-2.2.14 (Invitrogen) were used at a 1:1,000 and 1:2,500 dilution, respectively. AbcG1 polyclonal antibody (32) was used at a dilution of 1:500.

### Radial growth and drug disk-diffusion assay.

Fresh spores of A. fumigatus were suspended in 1× phosphate-buffered saline (PBS) supplemented with 0.01% Tween 20 (1× PBST). Spores in the suspension were enumerated using a hemocytometer to determine the spore concentration. Spores were then appropriately diluted in 1× PBST. For the drug diffusion assay, 1 × 10^6^ spores were mixed with 10 mL soft agar (0.7%) and poured over 15 mL regular agar containing (1.5%) minimal medium. A paper disk was placed on the center of the plate, and 10 μL of 1 mg/liter voriconazole was spotted onto the sterile filter paper. For the radial growth assay, ~100 spores (in 4 μL) were spotted on minimal medium with or without the drug. The plates were incubated at 37°C and inspected for growth every 12 h.

### Real-time PCR.

Reverse transcription-quantitative PCR (RT-qPCR) was performed as described in reference 19, with the following modification. Cell lysates were prepared from mycelial biofilm cultures formed upon inoculating 10^6^ spores in a petri dish containing 20 mL of Sabouraud dextrose broth and allowing growth for 24 h at 37°C under nonshaking conditions. The threshold cycle value of the *tef1* (*Afu1g06390*) transcript was used as a normalization control.

### Fluorescence microscopy.

To visualize the localization of NcaA-GFP2-5 and AtrR-mNeonGreen, conidia were inoculated in 200 μL of minimal medium on coverslips and incubated in moisture conditions at 37°C for 16 h (33). Coverslips were washed twice with 1× PBS and treated with 3 μg/mL Hoechst dye, a nuclear stain, for 15 min. Images were captured at 100× magnification using an Olympus fluorescence microscope BX60 controlled by iVision software (BioVision Technologies) and equipped with a Hamamatsu Orca-R2 digital camera. A GFP filter was used for visualization of tagged proteins with an excitation wavelength of 470 nm and emission wavelength of 509 nm. Excitation and emission wavelengths were 359 nm and 461 nm, respectively, for visualization of Hoechst staining. Adobe Photoshop 2022 was used for preparing images for publication.
